# Characterisation of cell functions and receptors in Chronic Fatigue Syndrome/Myalgic Encephalomyelitis (CFS/ME)

**DOI:** 10.1186/s12865-015-0101-4

**Published:** 2015-06-02

**Authors:** Sharni Lee Hardcastle, Ekua Weba Brenu, Samantha Johnston, Thao Nguyen, Teilah Huth, Naomi Wong, Sandra Ramos, Donald Staines, Sonya Marshall-Gradisnik

**Affiliations:** National Centre for Neuroimmunology and Emerging Diseases, Griffith Health Centre, School of Medical Science, Griffith University, Gold Coast, QLD Australia

**Keywords:** Chronic fatigue syndrome, Natural killer cell, Receptors, CD8^+^T Cell

## Abstract

**Background:**

Abnormal immune function is often an underlying component of illness pathophysiology and symptom presentation. Functional and phenotypic immune-related alterations may play a role in the obscure pathomechanism of Chronic Fatigue Syndrome/Myalgic Encephalomyelitis (CFS/ME). The objective of this study was to investigate the functional ability of innate and adaptive immune cells in moderate and severe CFS/ME patients. The 1994 Fukuda criteria for CFS/ME were used to define CFS/ME patients. CFS/ME participants were grouped based on illness severity with 15 moderately affected (moderate) and 12 severely affected (severe) CFS/ME patients who were age and sex matched with 18 healthy controls. Flow cytometric protocols were used for immunological analysis of dendritic cells, monocytes and neutrophil function as well as measures of lytic proteins and T, natural killer (NK) and B cell receptors.

**Results:**

CFS/ME patients exhibited alterations in NK receptors and adhesion markers and receptors on CD4^+^T and CD8^+^T cells. Moderate CFS/ME patients had increased CD8^+^ CD45RA effector memory T cells, SLAM expression on NK cells, KIR2DL5^+^ on CD4^+^T cells and BTLA4^+^ on CD4^+^T central memory cells. Moderate CFS/ME patients also had reduced CD8^+^T central memory LFA-1, total CD8^+^T KLRG1, naïve CD4^+^T KLRG1 and CD56^dim^CD16^−^ NK cell CD2^+^ and CD18^+^CD2^+^. Severe CFS/ME patients had increased CD18^+^CD11c^−^ in the CD56^dim^CD16^−^ NK cell phenotype and reduced NKp46 in CD56^bright^CD16^dim^ NK cells.

**Conclusions:**

This research accentuated the presence of immunological abnormalities in CFS/ME and highlighted the importance of assessing functional parameters of both innate and adaptive immune systems in the illness.

**Electronic supplementary material:**

The online version of this article (doi:10.1186/s12865-015-0101-4) contains supplementary material, which is available to authorized users.

## Background

The innate immune system exhibits rapid effector functions and acts as the first line of defence, protecting the host cells while activating the adaptive immune system. Natural killer (NK) cells in particular are an important interface for the innate and adaptive immune systems; hence, impaired function potentially leads to immunological disturbances. The presence of immunological dysfunction may impair physiological functioning and may play a role in disease pathogenesis [1]. NK cell dysregulation has been demonstrated in a number of illnesses, including human immunodeficiency syndrome (HIV), systemic lupus erythematosus (SLE), multiple sclerosis (MS) and Major Depressive Disorder (MDD) [2-4].

Patients with Chronic Fatigue Syndrome/Myalgic Encephalomyelitis (CFS/ME) exhibit reduced NK and CD8^+^T cell cytotoxic activity and differences in a number of adaptive immune cell phenotypes [5, 6]. Significant decreases in NK cell cytotoxic activity in CFS/ME patients who were moderately affected by symptoms and characterised using the Fukuda criteria for the illness have been extensively reported [5, 7-23]. The reduced NK cell cytotoxic activity in CFS/ME patients may be associated with abnormalities in NK cell phenotypes, receptors or lytic proteins. Of the previous CFS/ME studies, five have also found significant differences in perforin and granzymes in NK cells of CFS/ME patients [10, 20, 24-26]. These changes in functional and phenotypic components of immune cells may be playing a role in the pathomechanism of CFS/ME.

CFS/ME is an enigmatic illness which has no known pathomechanism or cause, with diagnoses based on symptom specific criteria and a range of exclusions. Due to the multifactorial and complex nature of CFS/ME, there are often misconceptions and inconsistencies surrounding diagnosis which highlights the importance of thorough screening of participants in both clinical and research settings. This is particularly important in determining those with other illnesses such as major depression who may satisfy the CFS/ME symptom specific criteria and also demonstrate NK cell abnormalities [2]. CFS/ME is characterised by persistent fatigue and a combination of symptoms which are often severely debilitating and the illness also tends to vary greatly in the nature of onset and symptom severity [5, 7, 27-31]. Moderate patients are mostly able to maintain normal daily activities but may be hampered by reduced mobility while severely affected CFS/ME patients experience high levels of daily fatigue and are typically housebound [32].

Importantly, severe CFS/ME patients’ NK cell cytotoxic activity has only been reported in three previous investigations [5, 7, 13]. Severely affected CFS/ME patients have demonstrated significant reductions in NK cell cytotoxic activity as well as increased NK cell receptor KIR3DL1 and enhanced plasma interleukin (IL)-4, tumour necrosis factor (TNF)-α and interferon (IFN)-γ [7, 13]. This research also suggested a correlation between low NK cell cytotoxic activity and severity of CFS/ME, based on clinical status [13]. As studies have investigated innate and adaptive immune cells in CFS/ME patients, it appears important to further examine functional parameters such as cell activity, receptors and adhesion molecules. Along with NK cell and CD8^+^T cell aberrations [5, 7, 11, 19, 33], dendritic cell (DC) phenotypes have been previously abnormal in CFS/ME patients [5] although to date no study has assessed DC activity in the illness. Similarly, iNKT cells are seldom examined although one study found differences in iNKT cell phenotypes in CFS/ME patients [5], suggesting the possibility of iNKT cell dysfunction in the illness and the need to assess cytotoxic granules in these cells. Cytotoxic granules have not previously been assessed in gamma delta (γδ) or regulatory T cells (Tregs) in CFS/ME patients, which may be cells of interest according to differences found in these cell types in previous research [10, 16, 17, 34].

Previous research has outlined the presence of NK cell dysfunction and immunological abnormalities in CFS/ME patients although most studies only assessed moderately affected CFS/ME patients. Immune dysregulation can also be associated with the clinical features and aetiology of CFS/ME. This is supported as research has found significant clinical improvements in CFS/ME patients’ symptoms after B cell depletion [35]. It is also possible that further immune cells may be affected by the illness and have yet to be assessed in CFS/ME patients. The current investigation was the first to measure the functional activity of DCs, neutrophils and monocytes, lytic proteins in iNKT, γδ and Tregs as well as receptors and adhesion molecules of NK, T and B cells in moderate and severe CFS/ME patients.

## Results

### No differences in participant characteristics between groups

Principal participant results are reported in Table 1. We found no statistical difference for age or gender between participant groups (*p* = 0.325, 0.607 respectively) (Table 1) and the Fukuda criteria for CFS/ME were satisfied by all moderate and severe CFS/ME participants.

**Table 1:** Participant characteristics and comparisons of the age (mean ± SEM) and gender distribution of each participant group (control, moderate and severe)

Parameter	Control (*n* = 18)	Moderate (*n* = 15)	Severe (*n* = 12)	*p* value
Age in years *(Mean ± SEM)*	40.39 ± 2.65	45.93 ± 2.96	41.25 ± 2.77	0.325
Gender Female, Male	12, 6	11, 4	10, 2	0.607

Age data is represented as Mean ± SEM in control (*n* = 18), moderate CFS/ME (*n* = 15) and severe CFS/ME (*n* = 12). *signifies *p* <0.05 between participant groups. There were no significant differences in age or gender within the research groups. *CFS/ME* Chronic Fatigue Syndrome/Myalgic Encephalomyelitis; standard error of the mean

### Severity scale scores differ between participant groups

In all severity scales used, including the Fatigue Severity Scale (FSS), Dr Bell’s Disability Scale, the FibroFatigue Scale and the Karnofsky Performance Scale (KPS), there were significantly different scores between all participant groups, with the exception of ‘sadness’ (*p* = 0.064). Moderate CFS/ME patient scores were significantly worsened compared with healthy controls in all parameters. Severe CFS/ME patients also displayed significantly worsened scores when compared with healthy controls, with the exception of ‘sadness’ (*p* = 0.194) and ‘sleep’ (*p* = 0.091). The KPS and Dr Bell’s Disability Scale scores were significantly lower in the severe CFS/ME patients compared with the moderate CFS/ME patients (*p* = 0.001 and 0.001).

### Differences in routine full blood count parameters between participant groups

**Fig. 1:**
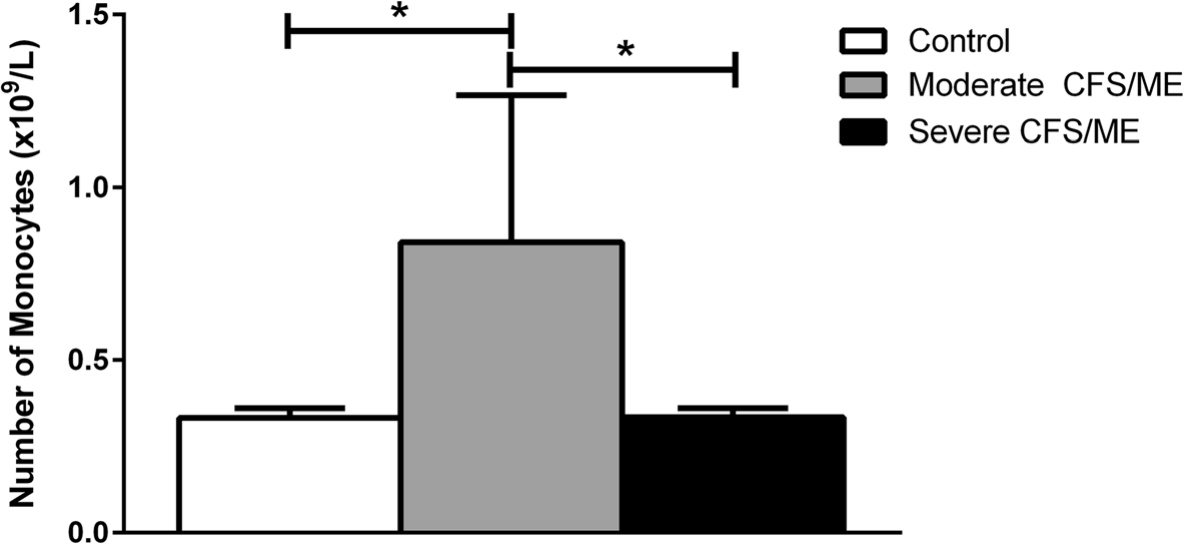
Monocyte full blood counts for controls, moderate and severe CFS/ME participants. Monocyte numbers (x10^9^/L) obtained from routine full blood counts are shown for controls, moderate and severe CFS/ME patients. Data is represented as mean ± SEM. *represents results that were significantly different where *p* <0.05. CFS/ME: Chronic Fatigue Syndrome/Myalgic Encephalomyelitis; SEM: Standard Error of the Mean

Our data have shown a significantly increased monocyte count in the moderate CFS/ME patients compared with the healthy controls and severe CFS/ME patients (Fig. 1). There were no other statistically significant differences in the routine full blood count parameters between the participant groups (data not shown).

### No differences in flow cytometric analysis of DC, neutrophil and monocyte function or lytic proteins

Previous research has reported differences in DC phenotypes in moderate and severe CFS/ME patients [5], however, this was the first research to assess DC activity in the illness. Our data have found no significant differences in the DC activity markers CD80 and CD86, in unstimulated or stimulated DCs between any of the participant groups, see Additional file 1: Figure S1. Neutrophil and monocyte function were examined as neutrophil respiratory burst has previously been reduced in moderate CFS/ME patients [8]. There were no significant alterations between any of the participant groups in the ability of neutrophils or monocytes to phagocytose or undergo respiratory burst, see Additional file 2: Figure S2. iNKT, γδT cells and Tregs have previously shown dysfunction in CFS/ME patients [5], however no studies had examined lytic proteins in these cell types. We found no significant differences in iNKT, γδT cells or Treg levels of perforin, granzyme A, granzyme B or CD57, see Additional file 3: Figure S3.

### NK cell adhesion molecules and natural cytotoxicity receptors differ between moderate and severe CFS/ME patients

**Fig. 2:**
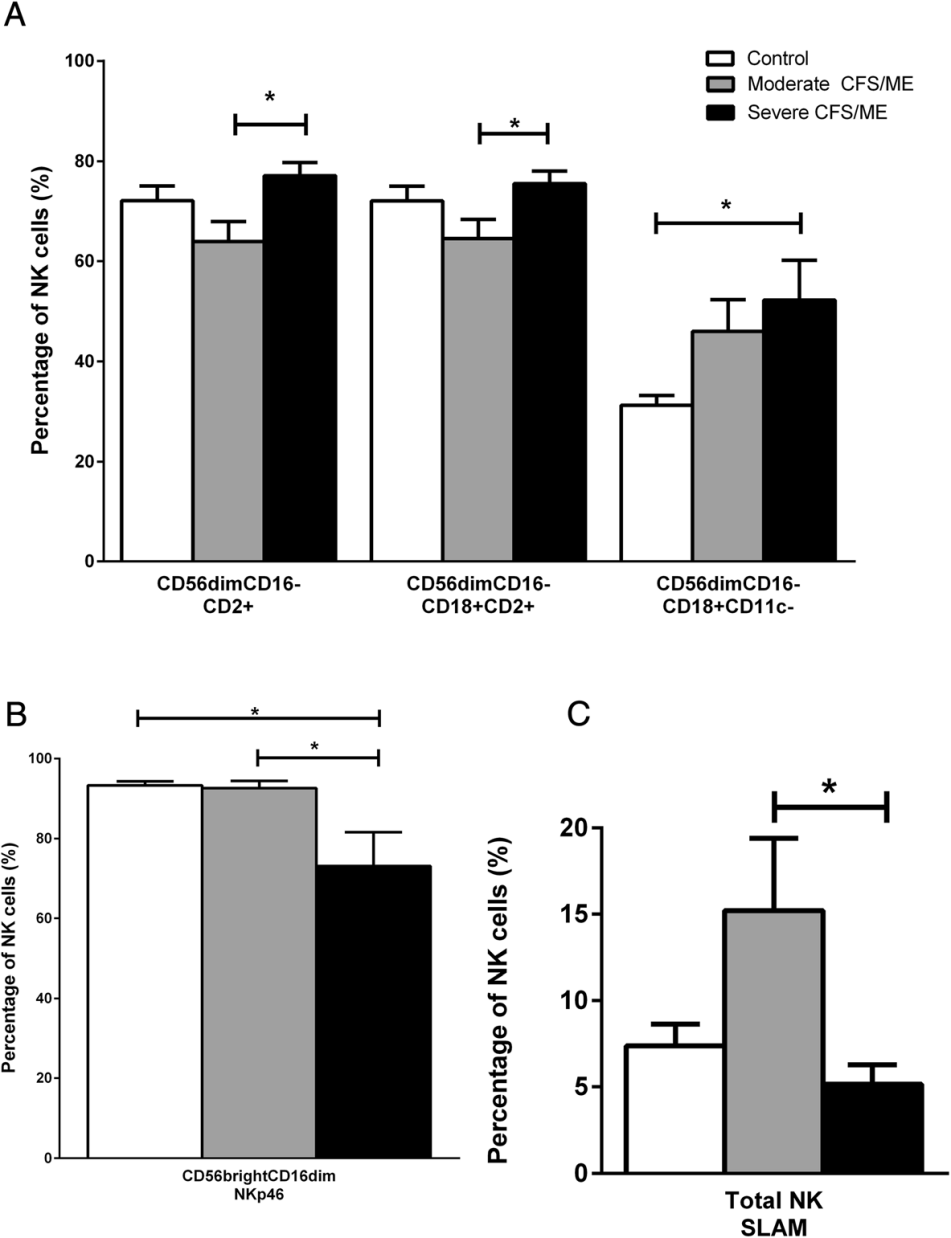
The profile of receptors and adhesion molecules on isolated NK cells in controls, moderate and severe CFS/ME participants. **a** Profile of CD2^+^, CD18^+^CD2^+^ and CD18^+^CD11c^−^ adhesion molecules on CD56^dim^CD16^−^ NK cells in control, moderate and severe CFS/ME patients. **b** CD56^bright^CD16^dim^ NK cell expression in control, moderate and severe CFS/ME patients, represented as percentage of NK cells. **c** Total NK cells expressing the SLAM receptor in control, moderate CFS/ME and severe CFS/ME patients. All data are represented as percentage of total NK cells (%) and shown as mean ± SEM. *represents results that were significantly different where *p* <0.05. NK: natural killer; CFS/ME: Chronic Fatigue Syndrome/Myalgic Encephalomyelitis; *SLAM*: signalling lymphocyte activation molecule; *SEM*: Standard Error of the Mean

Previous investigations have shown significant differences in NK cell receptors in CFS/ME patients, however signaling lymphocytic activation molecule (SLAM) receptors, adhesion molecules and natural cytotoxicity receptors have not been reported and are critical for NK cell function [5, 10]. SLAM receptor (CD150) was significantly increased in our data in total NK cells of moderate CFS/ME patients compared with severe CFS/ME patients (*p* = 0.046). CD56^bright^CD16^dim^ NK cells expression of NKp46 was significantly reduced in severe CFS/ME compared with controls and moderate CFS/ME (*p* = 0.021 and 0.021 respectively) (Fig. 2). CD56^dim^CD16^−^ NK cell CD2 expression was significantly lower in moderate CFS/ME compared with severe CFS/ME patients (*p* = 0.033) while CD18^+^CD2^−^ was increased in moderate CFS/ME patients compared with controls and severe CFS/ME in CD56^dim^CD16^−^ NK cells (*p* = 0.009 and 0.035 respectively). In the CD56^dim^CD16^−^ NK cells phenotype, CD18^+^CD11c^−^ was significantly increased in the severe CFS/ME compared with controls (*p* = 0.036) (Fig. 2) (Table 2).

**Table 2:** Summary of significant differences in parameters found between groups

	Significant Parameters	Group	Potential Result of Changes
NK cell	↓ CD56^dim^CD16^−^ CD2^+^	M v S	Impaired ability to adhere to target cells [24].
	↓ CD56^dim^CD16^−^ CD18^+^CD2^+^	M v S	Reduced NK cells in an active state hence lessened NK cell activation and cytotoxic activity [24, 55].
	↑ CD56^dim^CD16^−^ CD18^+^CD11c^−^	S v C	Decreased adhesive abilities or lower number of activated cells [55].
	↓ CD56^bright^CD16^dim^ NKp46	S v C, S v M	Reduced recognition and lysis of target cells [41].
	↓ Total SLAM	S v M	Lessened ability of NK cells to undergo cytotoxic activity [40].
CD4^+^ T cell	↑ Total KIR2DL5	M v S	Inhibition of T cell functions [51].
	↓ Naïve KLRG1	M v C, M v S	Enhanced T cell activation [46].
	↑ Central Memory BTLA4	M v C	Greater inhibitory signalling and modulation of immune responses [52].
CD8^+^ T cell	↑ CD45RA Effector Memory	M v C	Heightened capacity for cytotoxic activities [43].
	↓ Central Memory LFA-1	M v C	Lack of LFA-1 adhesion required for optimal cytotoxic activity [53].
	↓ Total KLRG1	M v C	Enhanced T cell activation [46].

↓ represents significant reductions and ↑ represents significant increases in the group listed first in the ‘Group’ column compared with the group listed second in the ‘Group’ column. *S* Severe CFS/ME patients; *M* Moderate CFS/ME patients; *C* Controls

### No differences in Bregs and BCRs

Significant B cell phenotypes have been reported in both moderate and severe CFS/ME patients [5], however, regulatory B (Breg) cells and B cell receptors (BCRs) in CFS/ME cohorts are yet to be examined [5, 35]. We found no significant differences in Breg cell phenotypes or BCRs between the participant groups, see Additional file 4: Figure S4.

### Increased KIR2DL5 in CD4^+^T cells of moderate CFS/ME patients

**Fig. 3:**
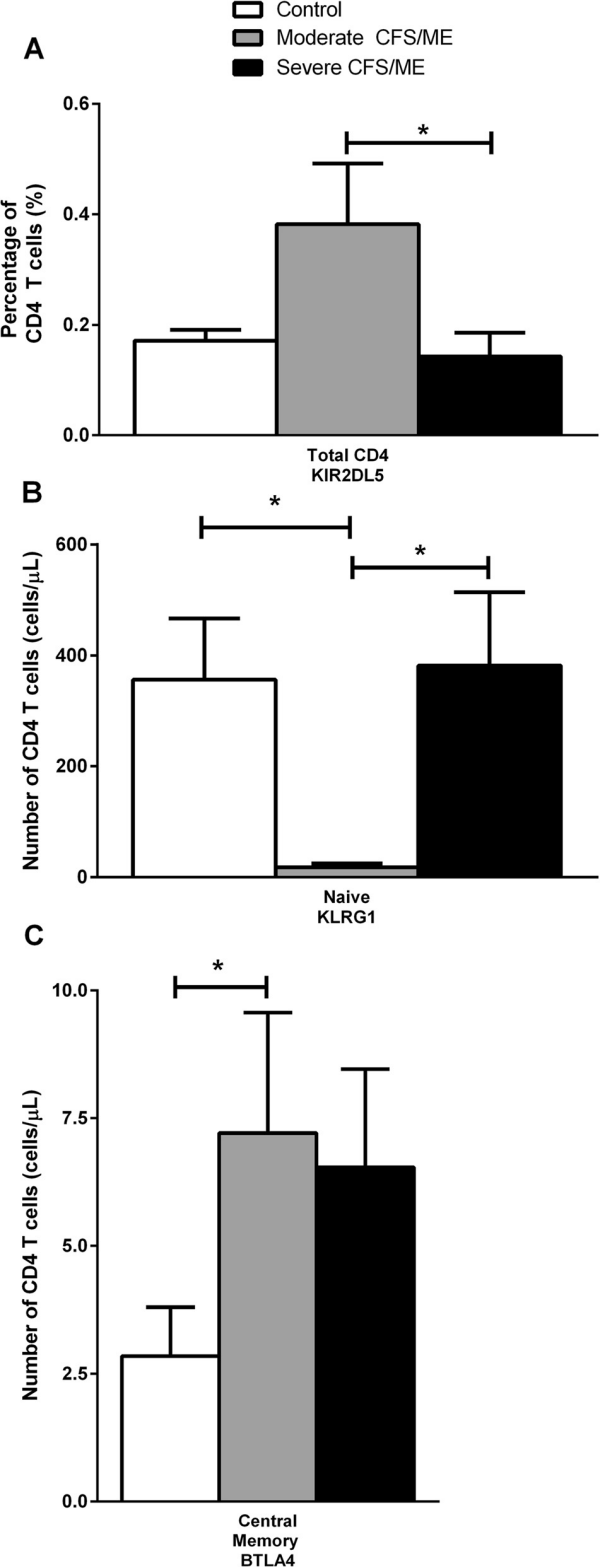
KIR and receptor expression in CD4^+^T cells of controls, moderate and severe CFS/ME participants. **a** KIR2DL5 expression in total CD4^+^T cells as represented by a percentage of total CD4^+^T cells (%). **b** Number of naïve CD4^+^T cells expressing KLRG1 in in control, moderate and severe CFS/ME participants shown as a number of cells per microliter (*cells/μL*). **C** BTLA4 receptor expression in central memory CD4^+^T cells of control, moderate and severe CFS/ME patients, presented as cells/μL. Data is represented as mean ± SEM. *represents results that were significantly different where *p* <0.05. KIR: killer immunoglobulin-like receptor; CFS/ME: Chronic Fatigue Syndrome/Myalgic Encephalomyelitis; KLRG1: killer cell lectin-like receptor subfamily G member 1; BTLA4: B and T lymphocyte attenuator SEM: Standard Error of the Mean

Killer immunoglobulin-like receptor (KIR)s have previously shown significant differences in NK cells of CFS/ME patients, although these had not been examined in CD4^+^T or CD8^+^T cells in CFS/ME patients [5, 7]. Our data found no significant alterations in the expression of KIRs on CD8^+^T cells between the participant groups. KIR2DL5 expression was significantly higher on CD4^+^T cells in moderate CFS/ME compared with severe CFS/ME patients (*p* = 0.011) (Fig. 3) (Table 2).

### Differences in CD8^+^T and CD4^+^T cells and phenotypes between CFS/ME patient groups

**Fig. 4:**
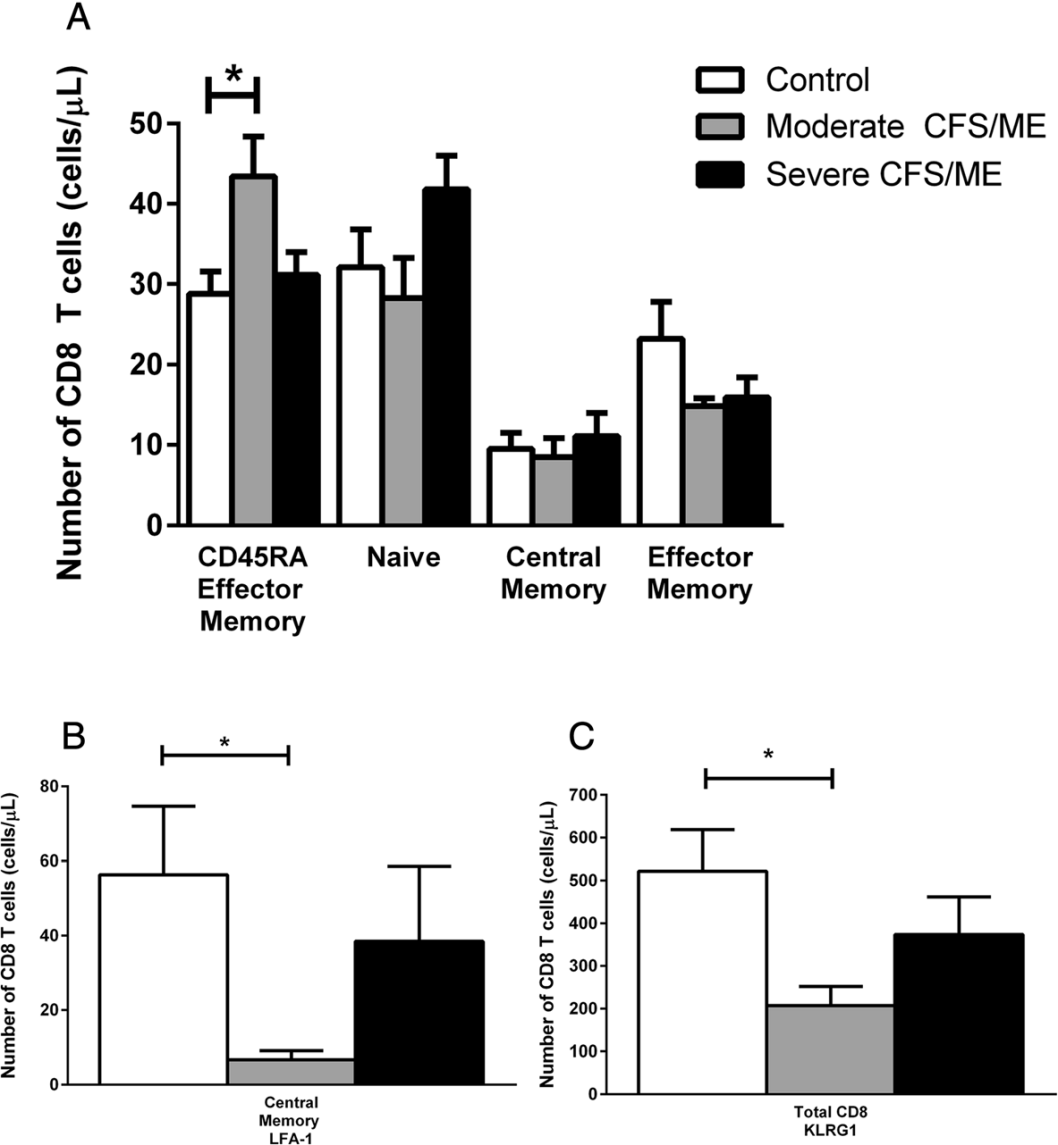
Alterations in peripheral blood CD8^+^T cell phenotypes and receptors in controls, moderate and severe CFS/ME. **a** CD45RA effector memory, naïve, central memory and effector memory CD8^+^T cell phenotypes represented as percentage of total CD8^+^T cells (%). **b** LFA-1 expression in central memory CD8^+^T cells for control, moderate CFS/ME and severe CFS/ME participants. **c** KLRG1 expression in total CD8^+^T cells for control, moderate CFS/ME and severe CFS/ME participants. All data are represented as mean ± SEM. * represents results that were significantly different where *p* <0.05. CFS/ME: Chronic Fatigue Syndrome/Myalgic Encephalomyelitis; LFA-1: lymphocyte function-associated antigen 1; KLRG1: killer cell lectin-like receptor subfamily G member 1; *SEM*: Standard Error of the Mean

CD8^+^T cells have been significantly different in CFS/ME patients in previous investigations, however, receptors on CD8^+^T and CD4^+^T cells had not yet been examined [6, 11, 25]. We found that the CD45RA effector memory CD8^+^T cell phenotype formed a significantly higher percentage of total CD8^+^T cells in moderate CFS/ME compared with controls (*p* = 0.016) (Fig. 4). Central memory CD8^+^T cells had significantly reduced lymphocyte function-associated antigen (LFA) -1 in moderate CFS/ME compared with controls (*p* = 0.032). Total CD8^+^T cell expression of killer cell lectin-like receptor subfamily G member (KLRG)1 was also reduced in moderate CFS/ME compared with controls (*p* = 0.014) (Fig. 4).

In our data, CD4^+^ central memory T cells, B and T lymphocyte attenuator (BTLA)4 had significantly increased expression in moderate CFS/ME patients compared with controls (*p* = 0.038) (Fig. 3). KLRG1 was also significantly reduced in CD4^+^ naïve T cells in moderate CFS/ME compared with controls and severe CFS/ME (*p* = 0.013 and 0.019 respectively) (Table 2). There was no significant difference in CD4^+^T cell phenotypes between any of the participant groups, see Additional file 5: Figure S5.

### Correlations across severity and immune parameters

Our data showed a significantly positive correlation between the KPS and Dr Bell’s Disability Scale scores. Both the KPS and Dr Bell’s Disability scale were negatively correlated with the total γδT cell CD45RA effector memory phenotype values and CD56^dim^CD16^−^ NK cells with CD18^+^CD11c^−^ (Table 3). There were also a number of parameters that were correlated with one another, such as CD4^+^T and CD8^+^T cell markers, NK cell adhesion markers and γδ and CD8^+^T cell phenotypes (Table 3).

**Table 3:** Spearman’s correlation to identify correlates between significant parameters

		1	2	3	4	5	6	7	8	9	10	11	12	13	14
1	CM CD8^+^ LFA-1														
2	Naïve CD4^+^ KLRG1														
3	Total CD8^+^ KLRG1		.50												
4	CM CD4^+^ BTLA4	.55													
5	CM CD4^+^ LFA-1			.56											
6	CD56^dim^CD16^−^ NK CD2^+^														
7	CD56^dim^CD16^−^ NK CD18^+^CD2^+^						-.60								
8	CD56^dim^CD16^−^ NK CD18^+^CD11a^−^		-.47		.49		-.57								
9	CD56^dim^CD16^−^ NK CD18^+^CD11c^−^														
10	Total NK SLAM			.46			-.53			-.50					
11	Total CD8^+^ EMRA			-.44		-.75									
12	CD4^+^ KIR2DL5								.48		.75				
13	KPS									-.43					
14	Dr Bell’s Disability Scale									-.46				.95	

Table 3 shows the significant correlation values between bivariate parameters for significant parameters for combined control, moderate CFS/ME and severe CFS/ME participant groups. All correlations shown have *p* < 0.01. CM: Central Memory, EMRA: CD45RA Effector Memory

## Discussion

This study is the first to provide an overview of cell function and receptor interactions, including assessing DC, neutrophil and monocyte function and receptors on T cells, BCRs and Bregs in moderate and severe CFS/ME patients. This is also the first study to examine lytic proteins in γδT cells, iNKT cells and Tregs in CFS/ME patients. Our results suggest that in some cases, functional immunological impairment may be related to differences in severity of CFS/ME patients, highlighting the variation in the illness. Significant alterations shown in moderate CFS/ME patients were often not present in severe CFS/ME patients and controls, revealing the possibility that moderate CFS/ME patients may have a different aetiology compared with the severely affected subgroup of CFS/ME patients.

Increased SLAM expression on total NK cells in moderate CFS/ME patients in this study may be a mechanistic response to the typically reduced NK cell cytotoxic activity in CFS/ME [10, 11, 36]. SLAM is a receptor expressed on the surface of T, B, NK and DC cells, functioning as an activating adaptor protein to amplify the recruitment of inflammatory cells, such as DCs, by activating IFN-γ [37-39]. SLAM receptors regulate NK cell activity via association with SLAM associated protein (SAP) family adapters, where binding receptors are then coupled to the Src kinase FynT to evoke protein tyrosine phosphorylation signals [40]. Heightened expression of SLAM in moderate CFS/ME may enhance the ability of NK cells to undergo cytotoxic activities [5, 7-11]. The activating receptor NKp46 is also typically involved in the recognition and lysis of target cells and was reduced in CD56^bright^CD16^dim^ NK cells of severe CFS/ME patients [41]. NKp46 is highly expressed on the CD56^bright^CD16^dim^ NK cell subset and although it is only weakly involved in cytotoxic activation, this reduction may contribute to the reduced NK cell cytotoxic activity prevalent in severe CFS/ME patients [5, 41, 42]. The expression of SLAM and NKp46 on NK cells significantly differed between moderate and severe CFS/ME patients, suggesting that perhaps these severity subgroups may vary in immunological presentation, as proposed by previous studies [5, 7, 13].

Effector memory and CD45RA effector memory cells demonstrate NK-like functions as they have the ability to detect abnormal major histocompatibility complex (MHC) expression and have a high capability for cytotoxic activities [43]. Our findings suggest that the number of CD45RA effector memory CD8^+^T cells of moderate CFS/ME patients may be enhanced due to sub-optimal function. In T cells, CD45RA effector memory cells are upregulated following cytokine directed proliferation, indicating that the subset is generated via homeostasis rather than antigen-dependent pathways [44, 45]. Moderate CFS/ME patients have an increased number of CD8^+^T CD45RA effector memory cells, potentially as a result of homeostasis where the cells may not be effectively undergoing degranulation and apoptosis [44, 45].

This research also found reductions in KLRG1 expression in total CD8^+^T and naïve CD4^+^T cells of moderate CFS/ME patients, which suggests that these cells may have a reduced ability to inhibit T cell function and activation. KLRG1 ligation inhibits the nuclear factor of activated T cells (NFAT) signalling pathway and downregulates CD95 mediated lysis to inhibit the activation of T cells [46]. Hence, blockades of inhibitory receptors tend to improve CD8^+^T cell responses by preventing inhibitory pathways [47]. It is therefore possible that reduced KLRG1 may be contributing to the pro-inflammatory response and T cell activation often found in CFS/ME patients [10, 48].

Increased KIR2DL5 on CD4^+^T cells in moderate CFS/ME patients may also be associated with alterations in KIR receptors in T cells in the same cohort [5, 7]. KIR2DL5 is an inhibitory KIR found in variable proportions of circulating T cells [49, 50] which is directly linked to a greater number of random combinations of KIR receptors expressed on these cells, which may be influencing optimal T cell functions in the illness [51]. Enhanced inhibitory signalling and modulation of immune responses are typical attributes of increased BTLA expression in T cells which may be present in moderate CFS/ME patients who have amplified expression of inhibitory receptor BTLA4 in central memory CD4^+^T cells [52]. Activation and function of CD4^+^T cells by NK cells is dependent on the engagement of the β_2_ integrin LFA-1. LFA-1 adhesion is necessary for optimal cytotoxic activity by both NK cells and CD8^+^T cells, also mediating NK cell degranulation via synergy with NKG2D [53]. Decreased expression of LFA-1 on central memory CD8^+^T cells in moderate CFS/ME patients suggests that there may be a lack of LFA-1 adhesion in CFS/ME which is required for ideal cytotoxic activity by NK cells and CD8^+^T cells [53]. Similar to the pattern shown in SLAM and NKp46 receptors on NK cells, CD45RA effector memory CD8^+^T cells and CD4^+^T and CD8^+^T cell receptors significantly differed between moderate and severe CFS/ME patients.

Cellular adhesion may be important in CFS/ME as it is required for target cell contact and NK cell effector function. Regulation of adhesion molecules is necessary for integrin target cell ligand interactions as the release of adherence results in lymphocyte movement [54]. CD2 expression in CD56^dim^CD16^−^ NK cells is reduced in moderate CFS/ME patients compared with severely affected patients, suggesting that these cells may have an impaired ability to adhere to target cells. This confirmed previous findings where CD2/CD18 co-expression was reduced in the same CD56^dim^CD16^−^ NK cell phenotype in a cohort of moderate CFS/ME patients [24]. Increased CD2 is often associated with a higher cytotoxic ability [55] as CD2 acts as a contributor to induce NK cell activation [54, 55]. Higher expression of CD2 in severe CFS/ME patients potentially implies that more NK cells in these patients are in an active state and may have a greater ability than the moderate CFS/ME patients to induce NK cell activation and cytotoxic activities [55]. CD18^+^/CD2^−^ CD56^dim^CD16^−^ NK cells were also increased in the moderate CFS/ME patients, strengthening the theory that CFS/ME patients may have a weakened ability to activate NK cells as well as having impaired NK cell cytotoxic activity. Adhesion molecules CD18 and CD2 on CD56^dim^CD16^−^ NK cells were also significantly altered in the moderate CFS/ME patient group compared with the severe CFS/ME patients, who appeared similar to the controls. In the case of CD18^+^CD11c^−^ on the same CD56^dim^CD16^−^ NK cell subset, however, increases in CD18^+^CD11c^−^ increased in the moderate CFS/ME patients and significantly increased in the severe CFS/ME patients. Expression of the adhesion marker CD11c is heterogeneous and variable in NK cells although, typically activated NK cells are CD11c^+^ [55]. Increased CD18^+^CD11c^−^ on CD56^dim^CD16^−^ NK cells in severe CFS/ME patients indicates that these patients may have a reduced ability to adhere or that they may have a low number of activated NK cells. Therefore, differences in CD18^+^CD11c^−^ adhesion molecules in severe CFS/ME patients may be associated with the reduced NK cell cytotoxic activity found in the illness [5, 10, 11].

CFS/ME symptom severity and presentation may be related to the immune dysregulation shown as the immune system interacts with physiological functioning via a number of body systems, including the central nervous system, digestive system and endocrine system [56]. Unrefreshing sleep and sleep disturbances are symptoms of CFS/ME and reports have indicated that NK cells are altered after sleep deprivation, demonstrating interactions between physiological symptoms and the immune system [57], particularly in CFS/ME patients. Similarly, it has previously been suggested that clinical severity status appears to be associated with reduced NK cell activity in CFS/ME patients [7, 13]. Although there are limited research findings for severe CFS/ME patients, the differences in NK cells, CD4^+^T and CD8^+^T cells between severity groups, found in this research, suggest that immune dysfunction in CFS/ME may be related to clinical symptoms and hence severity.

## Conclusions

This study was the first to show significant differences in a number of receptors in NK, CD4^+^T and CD8^+^T cells in CFS/ME [5, 7] suggesting dysregulation in NK cell cytotoxic activity, receptor regulation and potentially cell adherence. Consistent with previous literature, our research suggests that CFS/ME patients have immunological dysregulation in the innate and adaptive immune cells. We have also highlighted significant differences in NK, CD4^+^T and CD8^+^T cells between moderate and severe CFS/ME patients, suggesting severity subgroups may have distinct immune perturbations and consequently aetiology. Further studies examining severity subgroups of CFS/ME patients may therefore contribute to the understanding of the pathomechanism associated with the illness.

## Methods

### Participants

Participants aged between 20 and 65 years old were recruited from Queensland and New South Wales areas of Australia through CFS/ME support groups, email advertisements and social media. In the absence of a diagnostic test for CFS/ME, the 1994 Fukuda criteria were used and patients must have had the illness for a period of at least 6 months prior to the study. All CFS/ME patients had been diagnosed by a primary physician in order to take part in the research. CFS/ME patients were identified as either moderate (mobile) or severe (housebound). These severity groups were then confirmed using an extensive questionnaire which included FSS, Dr Bell’s Disability Scale, the FibroFatigue Scale and the KPS as determinants of severity. Participants were then excluded if they were previously diagnosed or had a history of an autoimmune disorder, MS, psychosis, major depression, heart disease or thyroid-related disorders or if they were pregnant, breast feeding, smokers, or experiencing symptoms of CFS/ME that did not conform to the Fukuda criteria for CFS/ME [5]. The questionnaire obtained detailed information regarding the onset of illness, presence, frequency and severity of symptoms, comorbidities, overall health and quality of life. This allowed an analysis of all participants on an individual basis to ensure there were no confounding factors influencing CFS/ME patient symptoms. In order to identify psychological exclusions in participants that were not specifically outlined, the questionnaire (including the FSS, SF-36 and WHO DAS2.0) also included questions and scales regarding emotional stability, social interaction, motivation and wellbeing [58, 59].

Participants (*n* = 45) included in the study were patients moderately (*n* = 15) or severely (*n* = 12) affected by CFS/ME symptoms as well as a healthy non-fatigued control group (*n* = 18). All participant groups were matched for age and sex. All CFS/ME patients had the illness for at least 6 months prior to their participation in the research and the average duration of illness of a CFS/ME patient was 6.5 years. It was then ensured that all participating CFS/ME patients fulfilled the Fukuda criteria for CFS/ME at the time of blood collection according to questionnaires which assessed their symptoms in the 30 days prior and at the time of collection. Written informed consent was obtained from all participants and all research protocols were granted ethical clearance after review by the Griffith University Human Research Ethics Committee (GU Ref No: MSC/23/12/HREC).

### Sample preparation and routine measures

Blood collection occurred between 8:00 and 11:30 am and samples were analysed within 12 h. A non-fasting blood sample of 50 mL was collected into lithium heparinised and ethylenediaminetetraacetic acid (EDTA) tubes from the antecubital vein of all participants. Initial full blood count results were determined by Pathology Queensland to assess routine levels of white blood cell and red blood cell markers.

### Lytic proteins analysis

Lytic proteins were assessed as previously described [7, 10] in Tregs, iNKT, γδ1 and γδ2 T cells. Ficoll-hypaque (Sigma, St Louis, MO) density gradient centrifugation was used to isolate peripheral blood mononuclear cells (PBMCs) from EDTA whole blood. PBMCs were adjusted to 1 × 10^7^ cells/mL and stained with monoclonal antibodies for iNKT, Tregs, γδ1 and γδ2 T cells, see Additional file 6: Table S1. Cells were incubated for 30 min in Cytofix then perforin, granzyme A and granzyme B monoclonal antibodies were added for 30 min. Cells were analysed on the flow cytometer where perforin, granzyme A and granzyme B expression was measured in iNKT, Tregs, γδ1 and γδ2 T cells, see Additional file 6: Table S1.

### DC cell activity analysis

DC activity was measured from 300uL lithium heparinised whole blood, incubated in Roswell Park Memorial Institute (RPMI)-1640 culture media (Invitrogen, Carlsbad, CA), Phorbol 12-myristate 13-acetate (PMA) and Ionomycin for 5 h at 37 °C, 5 % CO_2_. Cells were labelled with monoclonal antibodies (see Additional file 6: Table S1) and FACS Lyse (BD Biosciences, San Diego, CA) was used to remove red blood cells. Flow cytometric analysis (Becton Dickinson Immunocytometry Systems) was used to measure DC activity based on unstimulated versus stimulated assessments.

### Phagocytosis analysis

The Phagotest kit was used to determine leukocyte phagocytosis in whole blood based on the ability of neutrophils and monocytes to engulf bacteria (Orpegen Pharma, Germany). Manufacturer’s instructions were followed for the procedure. Lithium heparinised whole blood (100uL) was aliquot into two 5 mL tubes as control and test samples. Control and test samples were incubated with *E.coli* bacteria (Orpegen Pharma, Germany) on ice or at 37 °C respectively, before quenching solution were added to stop phagocytosis. Samples were washed and red blood cells were lysed with lysing solution (Orpegen Pharma, Germany). DNA staining solution was used as a measure of neutrophil and monocyte phagocytosis based on differential gating on a flow cytometric analysis (Becton Dickinson Immunocytometry Systems).

### Respiratory burst analysis

Respiratory burst analysis was measured in granulocytes from whole blood. Intracellular oxidation was performed by incubating 100uL lithium heparinised whole blood in Dihydrohodamine (DHR) for 10 min at 37 °C. Phorbol 12-myristate 13-acetate was then added, followed by 10 min incubation at 37 °C. FACS Lyse (BD Biosciences, San Diego, CA) was used to remove red blood cells and DHR was used as a measure of neutrophil and monocyte respiratory burst using differential gating on the flow cytometer (Becton Dickinson Immunocytometry Systems).

### Isolated NK cell receptor analysis

As previously described [5], NK cells were isolated from whole blood cells using negative selection with RosetteSep Human Natural Killer Cell Enrichment Cocktail (StemCell Technologies, Vancouver, BC). Isolated NK cells were labelled with CD56, CD16, CD3 (BD Biosciences, San Diego, CA) and monoclonal antibodies for SLAM, integrin and natural cytotoxicity receptors (NCRs), see Additional file 6: Table S1 (Miltenyi Biotec). Analysis was undertaken on the flow cytometer (Becton Dickinson Immunocytometry Systems), where NK cells were gated using CD56, CD16 and CD3 and SLAM, integrin and NCR receptors were assessed for each NK cell phenotype (CD56^dim^CD16^+^, CD56^bright^CD16^+^, CD56^dim^CD16^−^ and CD56^bright^CD16^dim^) see Additional file 6: Table S1 [7].

### T and B cell whole blood analysis

Whole blood analysis was undertaken as previously described [5]. Monoclonal antibodies were added to lithium heparinised whole blood samples and incubated for 30 min, see Additional file 6: Table S1. Cells were then lysed, washed and fixed. CD4^+^T and CD8^+^T cell KIR receptors and phenotypes, B cell receptors and B regulatory cells were assessed using appropriate antibodies (see Additional file 6: Table S1) and gating strategies on the flow cytometer (Becton Dickinson Immunocytometry Systems) [60].

### Statistical analysis

Data were compared among the three participant groups (control, moderate CFS/ME and severe CFS/ME) with statistical analysis performed based on the distribution. Shapiro-Wilk normality tests were performed and if normally distributed, the analysis of variance test (ANOVA) was used. If data was not normally distributed, the Kruskal Wallis test of independent variables based on rank sums to determine the magnitudes of group differences was used. The Bonferroni Post Hoc or Mann–Whitney U tests determined *p* values of significance for parametric and non-parametric data respectively, with statistical significance set at an alpha criterion at *p* < 0.05. Spearman’s non-parametric correlation was conducted on significant parameters to determine correlates where significance was accepted as *p* < 0.01. Outliers were identified using a boxplot technique and handled by eliminating particular extreme data points from the analysis [61].

SPSS statistical software version 22.0 was used for all statistical analysis and data represented in this study are reported as plus/minus the standard error of the mean (±SEM) or plus/minus the standard deviation (SD) as specified.

## Additional files

Additional file 1: Figure S1. Flow cytometric analysis of dendritic cell activity in controls, moderate and severe CFS/ME patients using CD80 and CD86 activation markers. DC activity is shown based on the difference in expression of activation markers (CD80 and CD86) following stimulation. Measures are represented as percentage of DCs expressing markers (%) in controls, moderate CFS/ME and severe CFS/ME. All data are represented as mean ± SEM. CFS/ME: Chronic Fatigue Syndrome/Myalgic Encephalomyelitis; DC: Dendritic Cell. SEM: Standard Error of the Mean. Additional file 2: Figure S2. Flow cytometric analysis of monocyte and neutrophil function in controls, moderate and severe CFS/ME patients. A Monocyte and Neutrophil function respiratory burst, shown as difference in mean fluorescence intensity following stimulation. Measures are shown for controls, moderate CFS/ME and severe CFS/ME. B Monocyte and Neutrophil function phagocytosis, shown as difference in mean fluorescence intensity following stimulation. Measures are shown for controls, moderate CFS/ME and severe CFS/ME. CFS/ME: Chronic Fatigue Syndrome/Myalgic Encephalomyelitis. Additional file 3: Figure S3. Intracellular analysis of lytic proteins in controls, moderate and severe CFS/ME patients. A Lytic proteins in iNKT cells are shown as percentage of iNKT cells (%) expressing perforin, granzyme A, granzyme B and CD57 for controls, moderate CFS/ME and severe CFS/ME patients. B Lytic proteins in Tregs are shown as percentage of Tregs (%) expressing the lytic proteins perforin, granzyme A, granzyme B and CD57 in controls, moderate CFS/ME and severe CFS/ME patients. All data are represented as mean ± SEM. Chronic Fatigue Syndrome/Myalgic Encephalomyelitis; Treg: T Regulatory Cell. SEM: Standard Error of the Mean. Additional file 4: Figure S4. Profile of Bregs and BCRs in controls, moderate and severe CFS/ME patients. A Bregs are as percentage of B cells (%) in controls, moderate CFS/ME and severe CFS/ME patients. B CD79b and IgD expression to represent BCRs and shown as a percentage of B. BCRs shown in controls, moderate CFS/ME and severe CFS/ME patients. All data are represented as mean ± SEM. Breg: B Regulatory Cell; BCR: B Cell Receptor; Chronic Fatigue Syndrome/Myalgic Encephalomyelitis; Treg: T Regulatory Cell. SEM: Standard Error of the Mean. Additional file 5: Figure S5. Peripheral blood CD4^+^T cell phenotypes in controls, moderate and severe CFS/ME patients. CD45RA effector memory, naïve, central memory and effector memory CD4^+^T cell phenotypes represented as percentage of total CD4^+^T cells (%). All data are represented as mean ± SEM. CFS/ME: Chronic Fatigue Syndrome/Myalgic Encephalomyelitis; SEM: Standard Error of the Mean. Additional file 6: Table S1. Monoclonal antibody combinations used to identify various innate and adaptive immune cells and phenotypes.

## Abbreviations

ANOVA: Analysis of variance test
BCR: B cell receptor
Breg: B regulatory cell
BTLA: B and T lymphocyte attenuator
CFS: Chronic fatigue syndrome
DC: Dendritic cell
DHR: Dihydrohodamine
EDTA: Ethylenediaminetetraacetic acid
KPS: Karnofsky performance scale
FSS: Fatigue severity scale
HIV: Human immunodeficiency syndrome
IFN: Interferon
IL: Interleukin
KIR: Killer immunoglobulin-like receptor
KLRG: Killer cell lectin-like receptor subfamily G member
LFA: Lymphocyte function-associated antigen
MDD: Major depressive disorder
ME: Myalgic Encephalomyelitis
MHC: Major histocompatibility complex
MS: Multiple sclerosis
NCR: Natural cytotoxicity receptor
NK: Natural killer
NFAT: Nuclear factor of activated t cells
PBMC: Peripheral blood mononuclear cell
PMA: Phorbol 12-myristate 13-acetate
RPMI: Roswell park memorial institute
SAP: SLAM associated protein
SD: Standard deviation
SEM: Standard error of the mean
SLAM: Signaling lymphocytic activation molecule
SLE: Systemic lupus erythematosus
TNF: Tumour necrosis factor
Treg: T regulatory cell
γδ: Gamma delta
