# Supplementary material for: Characterisation of cell functions and receptors in Chronic Fatigue Syndrome/Myalgic Encephalomyelitis (CFS/ME)
Source: BMC Immunol. 2015 Jun 2;16:35. doi: 10.1186/s12865-015-0101-4 (PMC4450981; doi:10.1186/s12865-015-0101-4)
Supplement: Additional file 6: Table S1. — Monoclonal antibody combinations used to identify various innate and adaptive immune cells and phenotypes. [file 12865_2015_101_MOESM6_ESM.docx]

**Additional Table 1:** **Monoclonal antibody combinations used to identify various innate and adaptive immune cells and phenotypes.**

| **Cell** | **Measured Phenotype** | **Monoclonal Antibody Marker Combinations** |
| --- | --- | --- |
| **NK Cells** | **Phenotypes**:  **Integrins**:  **SLAM:**  **NCRs:** | CD3^-^CD56^+/-^CD16^+/-^  CD2, CD18, CD11b, CD11c  CD150,  NKp30, NKp44, NKp46, NKp80 |
| **CD8^+^T and CD4^+^T cells** | **CD8**  **CD4**  **Phenotypes**  **KIRs**  **Receptors and Markers:** | CD8^+^CD3^+^  CD4^+^CD3^+^  Naïve: CD45RA^+^CD27^+^  Central Memory: CD45RA^-^CD27^+^  Effector Memory: CD45RA^-^CD27^-^  CD45RA^+^ Effector Memory: CD45RA^+^CD27^-^  KIR2DL1 (CD158a), KIR3DL1 (CD158e), KIR2DL2/DL3 (CD158b), KIR2DS4 (CD158i), KIR2DL1/DS1 (CD158a/h), KIR3DL1/DL2 (CD158e/k), KIR2DL5 (CD158f), NKG2D (CD314), NKG2 (CD94)  PD1, CD160, TIM3, 2B4, CD44, PSGL, CD62L, CXCR3, CD49d/CD29, LFA-1, KLRG1, CD127, BLTA4, CTLA4, Tregs (CD25^+^CD28^+^CD56^+^), CCR5, CD28, CCR7 |
| **iNKT Cells** | **Lytic Proteins:** | 6B11^+^CD3^+^  Perforin, GranzymeA, GranzymeB |
| **Tregs** | **Lytic Proteins:** | CD127^low^CD25^+^CD3^+^CD4^+^  Perforin, GranzymeA, GranzymeB |
| **γδ T cells** | **γδ 1 T cells:**  **γδ 2 T cells:**  **Phenotypes:**  **Lytic Proteins:** | γδ 1^+^CD3^+^CD45RA^+/-^CD27^+/-^  γδ 2^+^CD3^+^CD45RA^+/-^CD27^+/-^  Naïve: γδ^+^CD3^+^CD45RA^+^CD27^+^  Central Memory: γδ^+^CD3^+^CD45RA^-^CD27^+^  Effector Memory: γδ^+^CD3^+^CD45RA^-^CD27^-^  CD45RA^+^ Effector Memory: γδ^+^CD3^+^CD45RA^+^CD27^-^  Perforin, GranzymeA, GranzymeB |
| **DCs** | **Phenotypes:** | CD14^-^CD16^+^ DCs: Lin2^-^HLA-DR^+^CD16^+^  pDCs: Lin2^-^HLA-DR^+^CD123^+^  mDCs: Lin2^-^HLA-DR^+^CD33^+^ |
| **B Cells** | **BCRs:**  **Breg:** | CD19^+^  CD79a, CD79b, IgA, IgE, IgD, IgM, CD154,  CD27^-^CD19^+^CD5^+^CD1d^+^CD81^+/-^, CD21^+/-^ |

Additional Table 1 shows the monoclonal antibody combinations used to identify each of the cells and parameters for gating on the flow cytometer.
